# Electric‐Current‐Induced Phase Transformation in Cu_6_Sn_5_ Below Its Equilibrium Transition Temperature

**DOI:** 10.1002/advs.75499

**Published:** 2026-05-08

**Authors:** Shih‐kang Lin, Shubhayan Mukherjee, Yu‐chen Liu, Jun Mizuno

**Affiliations:** ^1^ Department of Materials Science and Engineering National Cheng Kung University Tainan Taiwan; ^2^ Core Facility Center National Cheng Kung University Tainan Taiwan; ^3^ Center For Resilience and Intelligence on Sustainable Energy Research (RiSER) National Cheng Kung University Tainan Taiwan; ^4^ Academy of Innovative Semiconductor and Sustainable Manufacturing National Cheng Kung University Tainan Taiwan; ^5^ Department of Mechanical Engineering National Cheng Kung University Tainan Taiwan

**Keywords:** Cu_6_Sn_5_, electric‐current‐induced phase transformation, electromigration, intermetallics, phase stability, synchrotron x‐ray diffraction

## Abstract

As semiconductor interconnects scale toward sub‐2 nm nodes, they are subjected to increasingly high current densities that challenge materials reliability. While phase transformations are traditionally interpreted through thermal equilibrium, electric current can introduce additional nonequilibrium effects that influence structural evolution. Here, we report a current‐driven monoclinic‐to‐hexagonal transformation in Cu_6_Sn_5_ occurring under a measured bulk temperature of ∼120°C, below the equilibrium η′ ↔ η transition temperature of 186°C–189°C. Using an ex situ synchrotron X‐ray diffraction series on separate current‐stressed samples together with transmission electron microscopy, we show that matched bulk‐temperature thermal aging alone did not reproduce the same transformation within the examined time window, whereas current stressing progressively converted η′‐Cu_6_Sn_5_ to η‐Cu_6_Sn_5_. The transformed state also exhibits a higher measured indentation modulus and hardness than the monoclinic reference under the present test conditions. These results demonstrate that electric current can drive unconventional structural evolution in Cu_6_Sn_5_ below the equilibrium transition temperature and provide a basis for understanding current‐assisted phase stability in conductive intermetallics.

## Graphical abstract

Electric current induces a monoclinic‐to‐hexagonal phase transformation in Cu_6_Sn_5_ at a measured bulk temperature of ∼120°C, below the equilibrium transition temperature. Ex situ synchrotron x‐ray diffraction, TEM, and matched thermal controls show that current stressing promotes the formation of a retained hexagonal η‐phase post‐stress state not reproduced by bulk thermal exposure alone.

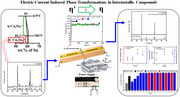

## 1 Introduction

The manipulation of phase transitions in condensed matter has historically relied on thermal or baric stimuli. However, as electronic systems reach the sub‐2 nm scale, high‐density current flow can introduce nonequilibrium effects that may influence defect behavior, diffusion, and phase stability under sub‐equilibrium thermal conditions [1, 2]. Rather than assuming that structural evolution under current can be described solely by bulk temperature, it is important to examine how electric current may modify transformation behavior under sub‐equilibrium thermal conditions [3, 4].

**FIGURE 1:**
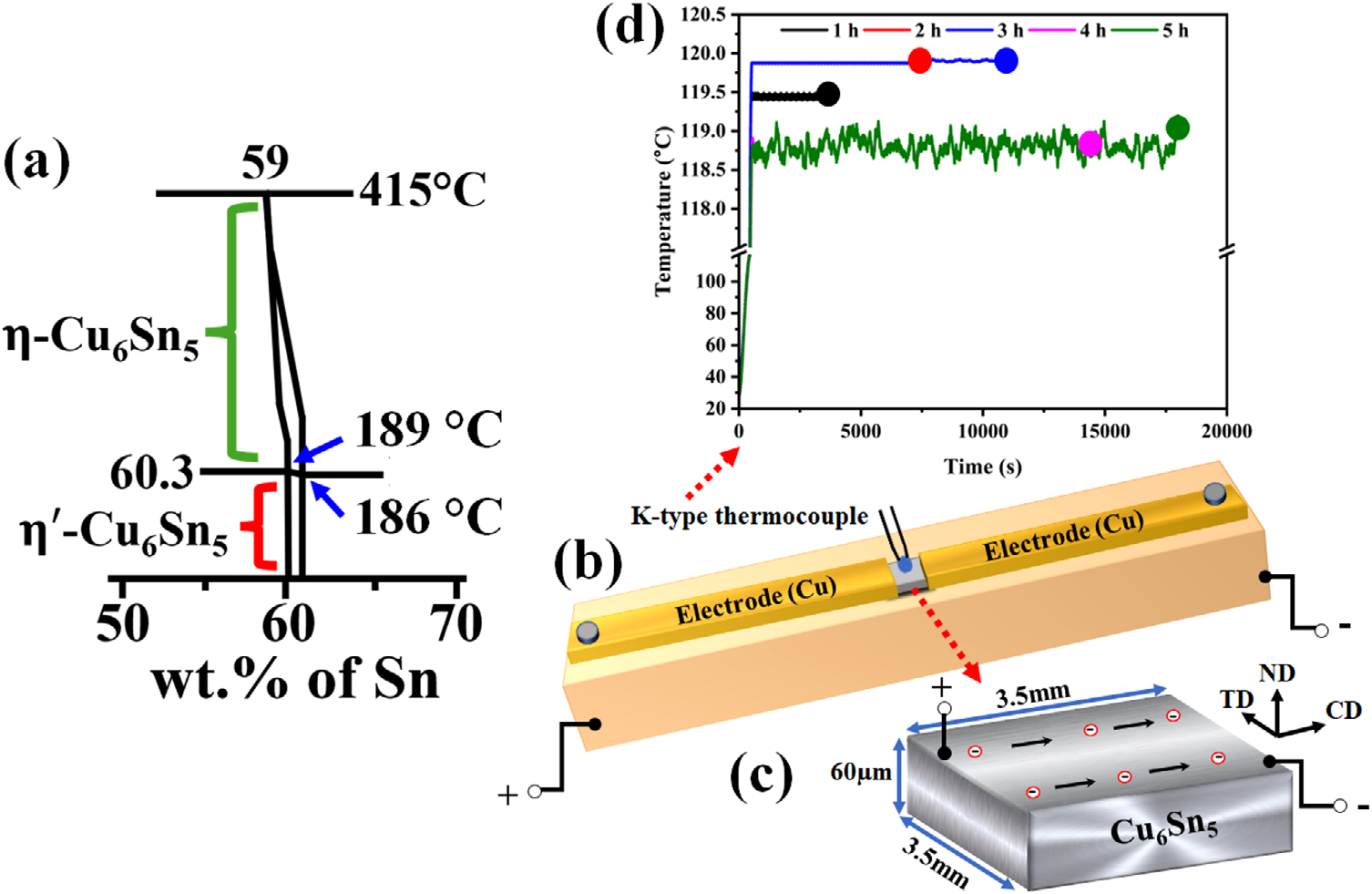
(a) Partial Cu–Sn binary phase diagram (50–70 wt.% Sn) highlighting the stability ranges of η′‐Cu_6_Sn_5_ and η‐Cu_6_Sn_5_ phases [8, 9, 10, 11, 12, 14, 17, 19, 27, 28]. (b) Experimental setup for electric‐current stressing, showing Cu electrodes and thermocouple position. (c) Geometry of the Cu_6_Sn_5_ specimen and the sample reference frame, where CD, TD, and ND denote the current, transverse, and normal directions, respectively. (d) Representative temperature profiles measured during current stressing for different stressing durations.

Soldering, also known as the reflow process, is the most important heterogeneous integration process in advanced electronic packaging. Sn‐based alloys are the major solder materials, and Cu is the most used substrate in electronic packaging. During soldering, interfacial reactions take place between Sn‐based solders and Cu substrates. The Cu_6_Sn_5_ phase is a main intermetallic compound (IMC) formed at the joints [5]. In micro‐bumps of 3D integrated circuits (3D ICs), the Sn‐rich body‐centered tetragonal (bct)‐(Sn) phase could be completely consumed, and results in the “full‐IMC” joints [6]. The interfacial IMCs critically determine the properties and reliability of electronic joints [7], not mentioning their role in full‐IMC joints. As shown in Figure 1a, the Cu_6_Sn_5_ phase exists in two polymorphs: the high‐temperature hexagonal η phase (space group P6_3_/mmc) and the low‐temperature monoclinic η′ phase (space group C2/c) [8, 9, 10]. Historically, the reversible η′ ↔ η transition was reported to occur near 186 °C [9, 10, 11, 12, 13], but subsequent refinements have shifted this transition slightly upward. More recent thermodynamic reassessments and high‐resolution diffraction studies have established a narrow but persistent range of 186°C–189°C for this polymorphic transformation [8, 14, 15, 16, 17, 18]. Leineweber et al., further noted that, at certain off‐stoichiometric compositions, the monoclinic structure can exhibit an incommensurate modulation, referred to as η′′, which is closely related to the commensurately ordered η′ superstructure [17, 19]. This shift in how the η′ ↔ η transition is described mirrors the steady improvement in characterization and thermodynamic modeling techniques over the past several decades. Beyond bulk assessments, laser‐heating experiments on Sn/Cu and Sn–0.1AlN/Cu stacks have recently clarified the early formation and growth of thin Cu_6_Sn_5_ films under short, high‐gradient thermal cycles [20]. Moreover, in situ diffraction/thermal‐expansion work has shown that the η′ ↔ η transformation is accompanied by a measurable lattice‐volume change and anisotropic expansion, which, under lateral constraint, induces compressive strain and interface roughening with reliability consequences [10, 12, 17, 21]. Larsson et al., further characterized this transformation, revealing modulated structures and twinning phenomena at elevated temperatures [22, 23]. Nogita et al., demonstrated that these two phases possess markedly different mechanical properties [24]. The hexagonal η‐Cu_6_Sn_5_ phase exhibits higher atomic density and higher crystal symmetry, resulting in higher ductility and hardness. In contrast, the monoclinic η′ phase is mechanically softer but also more brittle due to its lower symmetry and lattice distortion [25, 26]. These differences become especially critical in full‐IMC joints, where the absence of ductile Sn‐rich bct phase places greater mechanical reliability demands on the IMCs. The increased brittleness of η′‐Cu_6_Sn_5_ can elevate the risk of crack initiation and propagation under thermal or mechanical fatigue, whereas the more ductile η phase may better accommodate stress, improving joint reliability [21, 25].

In microelectronic interconnects, electric current induces a range of physical effects beyond simple Joule heating, significantly impacting IMC behaviors. Electromigration (EM), the drift of atoms under the electron wind force, is a primary reliability concern. Huntington and Grone first established this mechanism, showing that momentum transfer from electrons drives directional atomic diffusion and void formation, characterized by the “effective charge (z^*^)” [29]. Blech later demonstrated that such mass transport induces stress accumulation in thin films [30], while Conrad et al., found that current pulses reduce flow stress and alter dislocation behavior, evidencing electroplastic (EP) effects [31, 32]. Liu et al., used machine learning to predict z* values by linking them to elemental properties [33]. Chen et al., extended these classical findings to interfacial systems, where the polarity of current flow causes asymmetric IMC growth [5, 34, 35, 36]. Tu et al., showed enhanced Cu_6_Sn_5_ formation at the anode and suppression at the cathode, emphasizing the kinetic directionality of current‐driven diffusion [37, 38, 39, 40]. Recent in situ synchrotron X‐ray diffraction experiments revealed the “electric current‐induced lattice strain”, and the associated abnormal grain coarsening, recrystallization, and formation of peculiar grain morphology [41, 42, 43]. For full‐IMC joints, e.g., Cu/IMC/Cu, it is shown with superior EM resistance, with suppressed void formation and improved structural stability under high current densities, compared to conventional solder joints with remaining Sn‐rich bct phase [44, 45, 46]. Beyond promoting atomic diffusion and defect formation, Dolinsky and Elperin's early theoretical work demonstrated that current alters the thermodynamics of phase transitions and nucleation by modifying the Gibbs free energy landscape [47, 48, 49, 50]. This concept has since been extended through computational thermodynamics with the ab initio‐aided CALPHAD method by Lin et al., who reported that electric currents induce phase boundary shifts in the phase diagram of the Pb‐Sn binary system [51]. Recent comprehensive reviews summarize these phenomena, reinforcing that electric currents not only accelerate diffusion but also change the phase stability [41, 52].

However, despite increasing interest in current‐induced materials behavior, the influence of electric current on phase stability in conductive intermetallics remains insufficiently understood. In this study, we investigate whether Cu_6_Sn_5_ can undergo a monoclinic‐to‐hexagonal transformation during current stressing under a measured bulk temperature well below the equilibrium transition temperature. Using laboratory XRD, an ex situ synchrotron XRD series of separately current‐stressed samples, EBSD, and TEM/SAED, we compare matched bulk‐temperature thermal ageing with electric‐current stressing and evaluate the associated phase evolution, defect signatures, and mechanical response.

## 2 Results

**FIGURE 2:**
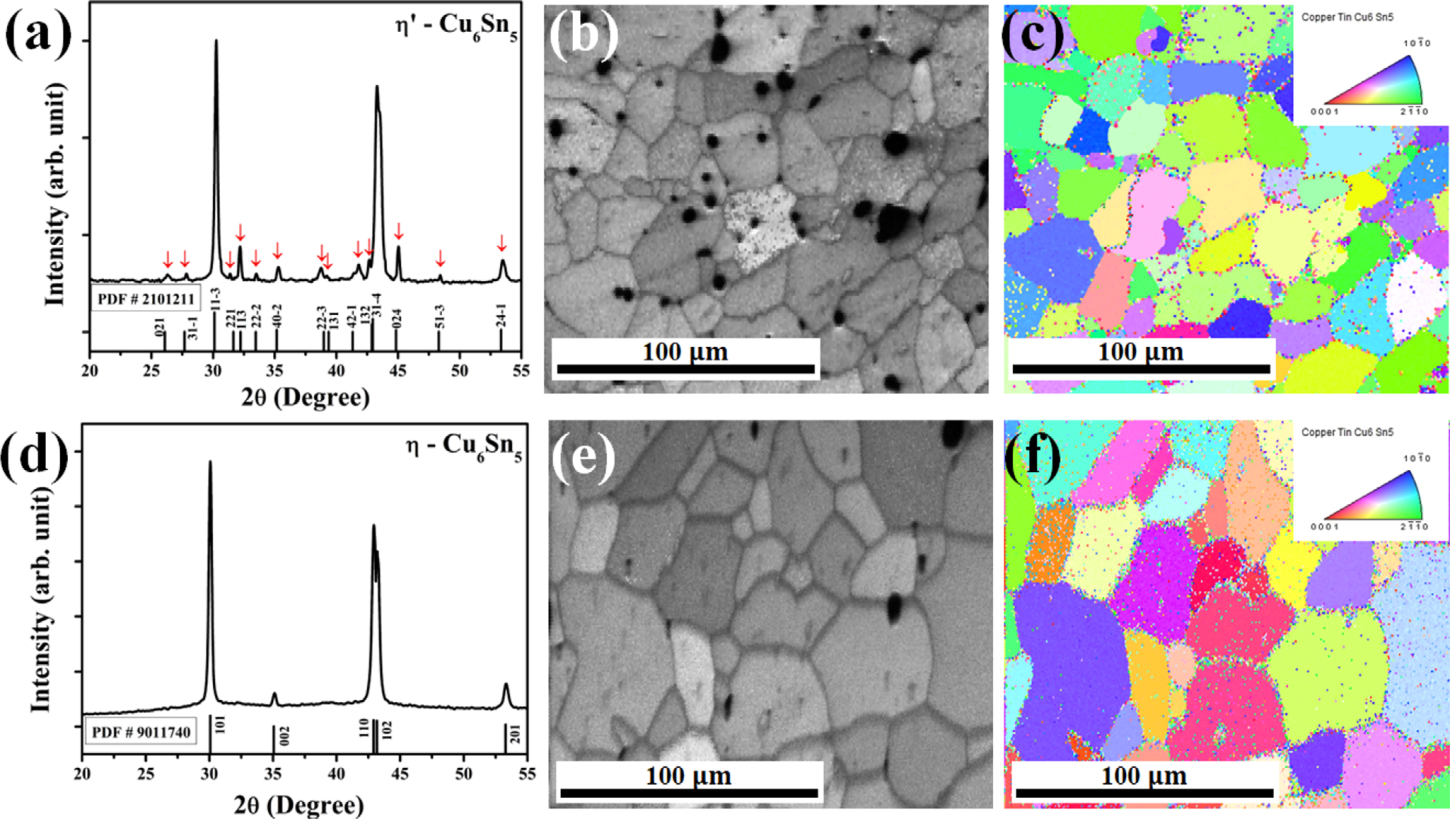
(a,d) The XRD patterns of as‐prepared monoclinic η′‐Cu_6_Sn_5_ and hexagonal η‐Cu_6_Sn_5_ phases, respectively, showing characteristic diffraction peaks based on PDF #2101211 and #9011740. (b,e) EBSD image‐quality (IQ) maps of the as‐prepared η′ and η states. (c,f) Corresponding EBSD inverse pole figure (IPF) maps referenced to the sample normal direction (ND).

Figure 2a,d show laboratory XRD patterns of Cu_6_Sn_5_ annealed for 24 h in vacuum at 150°C and 300°C, respectively, producing monoclinic η′‐Cu_6_Sn_5_ and hexagonal η‐Cu_6_Sn_5_ reference states. The weak reflections marked by arrows in Figure 2a are characteristic of η′‐Cu_6_Sn_5_ [53]. In contrast, the 300°C annealed sample matches the η‐Cu_6_Sn_5_ pattern (Figure 2d). Figure 2b,e present EBSD image‐quality (IQ) maps, and the corresponding inverse pole figure (IPF) maps (Figure 2c,f) are taken from the same fields of view. The IPF coloring is referenced to the sample normal direction (ND). These maps highlight the grain‐orientation contrast of the two reference states; the average grain sizes are 10.4 ± 0.6 µm for the η′ reference and 20.8 ± 0.5 µm for the η reference. Representative BSE images from the same samples (not the identical EBSD‐mapped regions) are provided in Figure S4 and show a uniform matrix with no obvious second‐phase contrast under the imaging conditions. Because η and η′ cannot be reliably separated by conventional Hough‐based EBSD indexing, all EBSD maps are indexed using the η phase library and are used only to describe grain morphology and orientation, while structural assignment (η′ vs η) is determined from XRD. Standards‐calibrated EDS (15 kV) gives Cu = 39.09 ± 0.3 wt.% and Sn = 60.91 ± 0.25 wt.%, consistent with stoichiometric Cu_6_Sn_5_ (Table S1).

**FIGURE 3:**
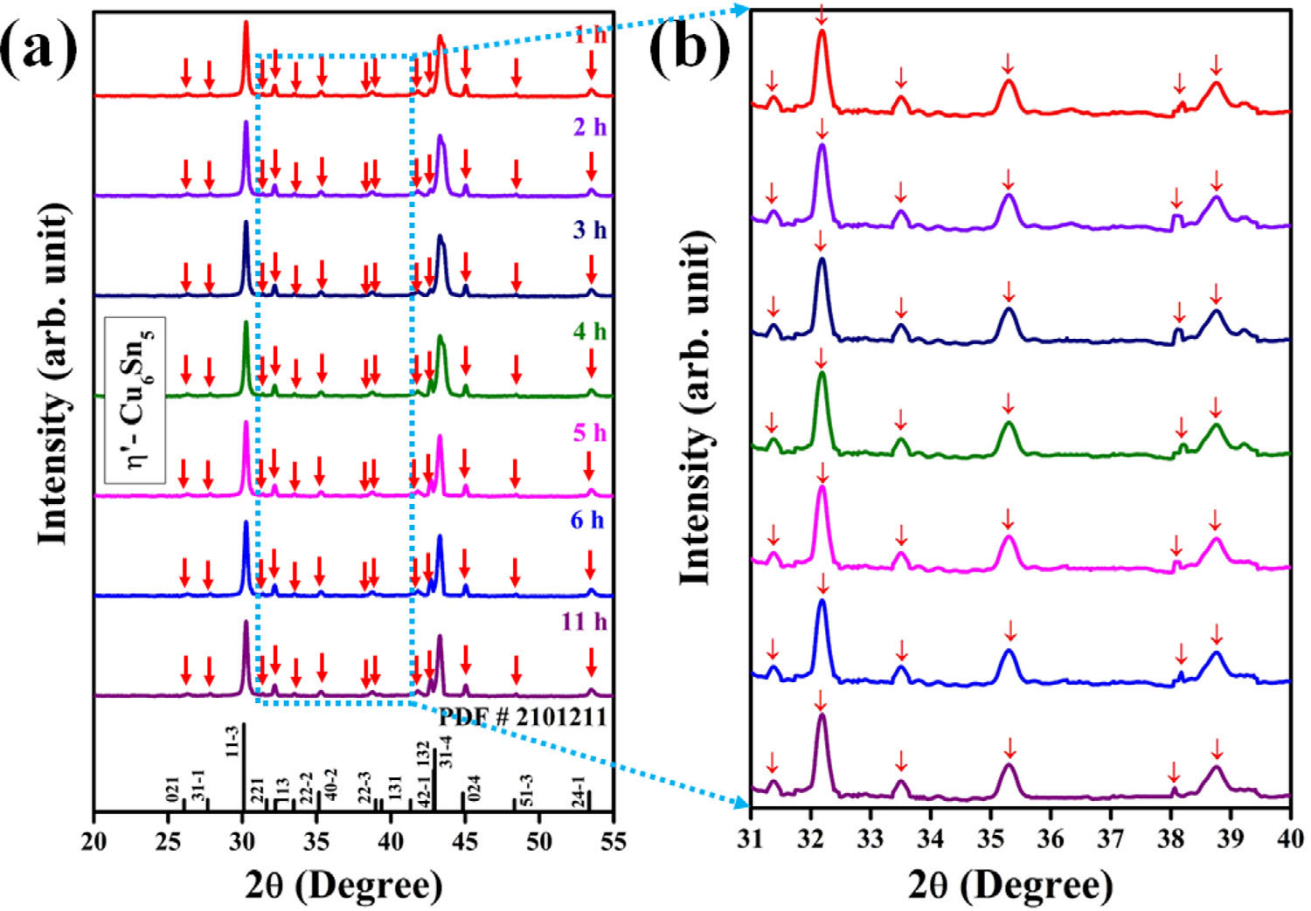
(a)The XRD patterns of the η′‐Cu_6_Sn_5_ samples aged at 120°C for 1, 2, 3, 4, 5, 6, and 11 h (the η′_1_, η′_2_, η′_3_, η′_4_, η′_5_, η′_6_, and η′_11_ samples). (b) Magnified XRD patterns of the region highlighted by the blue dotted box in (a), showing weak diffraction peaks (red arrows) corresponding to the monoclinic η′‐Cu_6_Sn_5_ phase.

**FIGURE 4:**
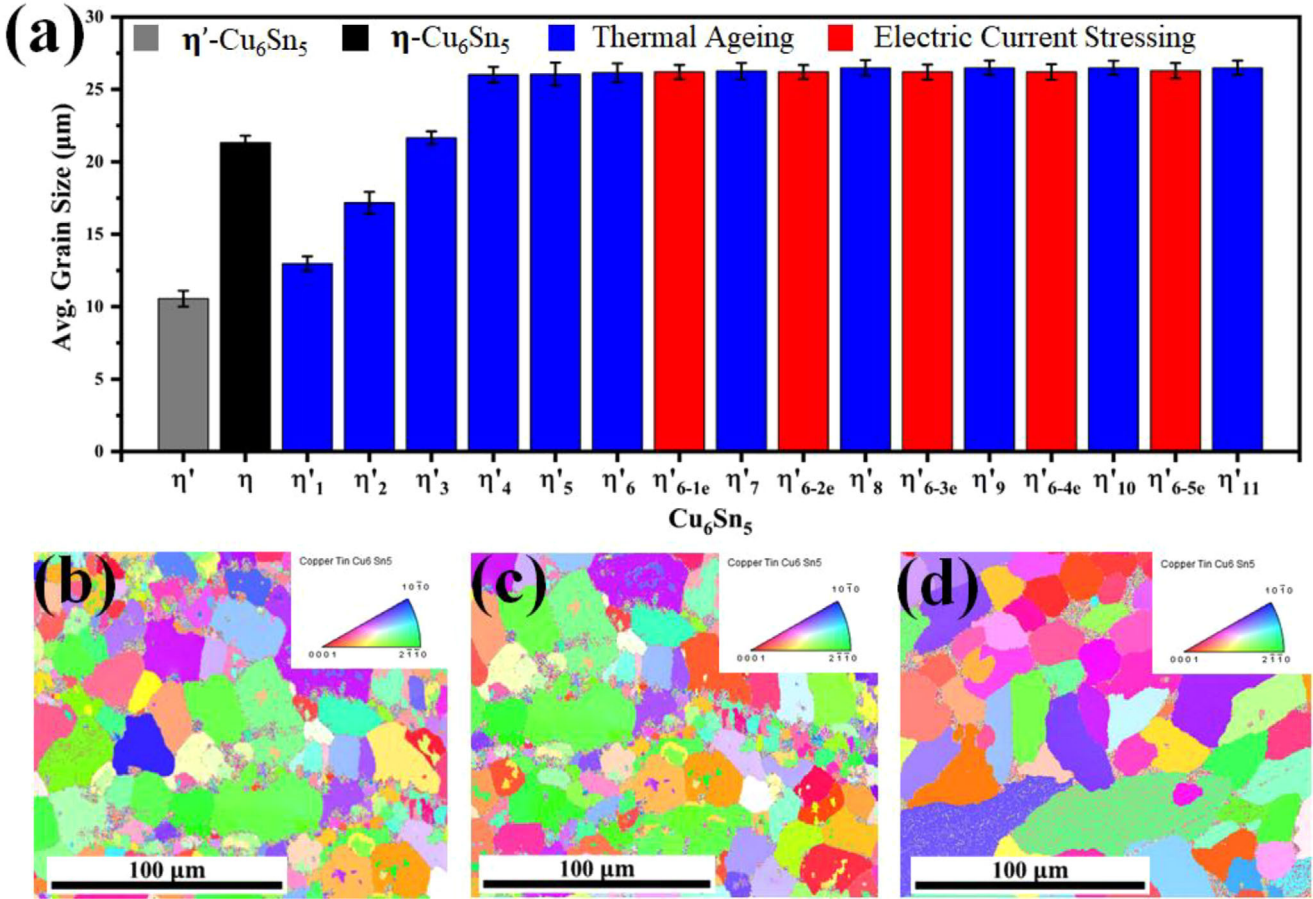
(a) Average grain size of the thermally aged and electrically stressed Cu_6_Sn_5_ samples. (b–d) EBSD inverse pole figure (IPF) maps referenced to the sample normal direction (ND) for η′_6_, η′_11_, and η′_6−5e_, respectively.

**FIGURE 5:**
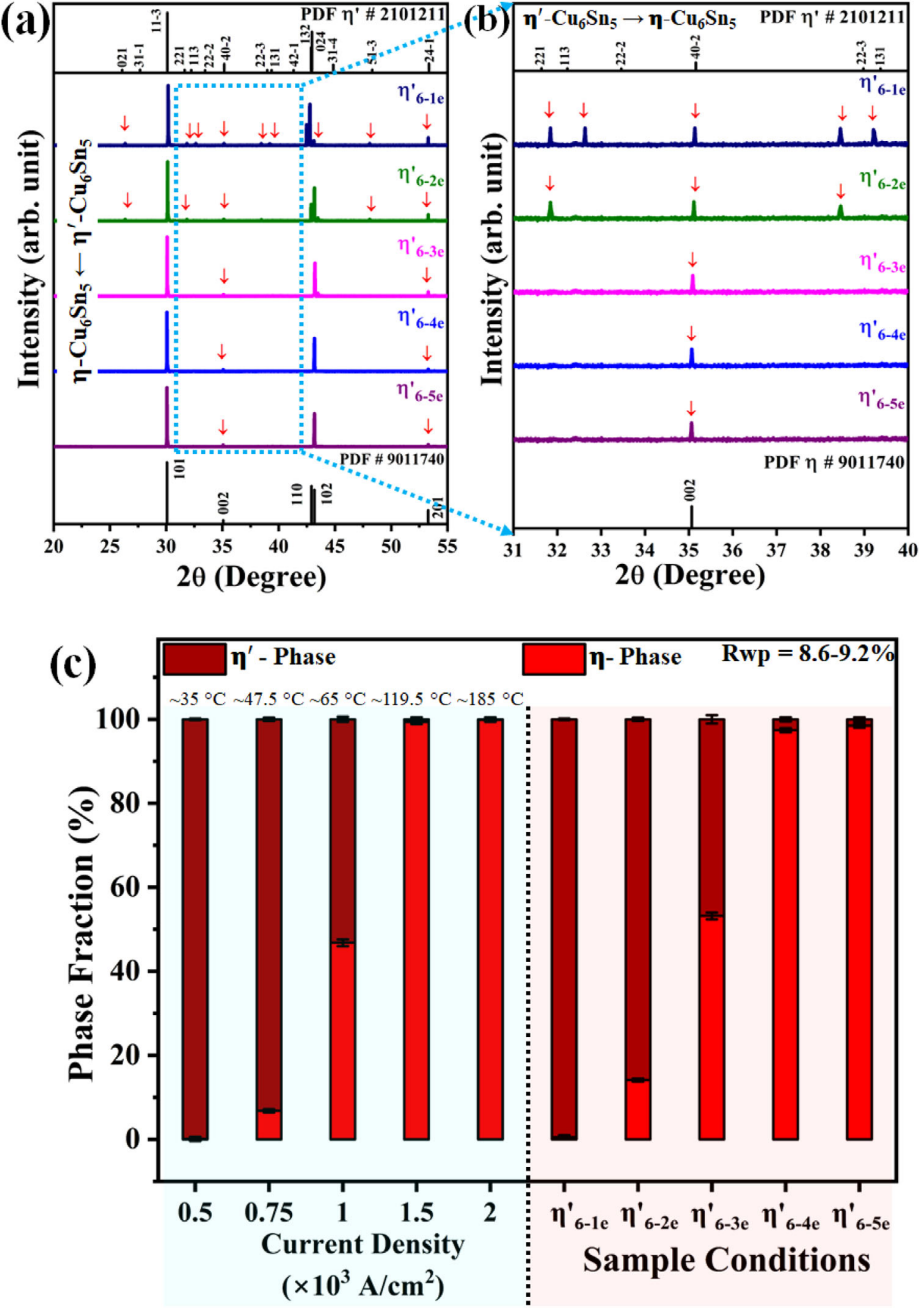
(a) SR‐XRD crystallographic observations of η′_6–1e_, η′_6–2e_, η′_6–3e_, η′_6–4e_, η′_6–5e_ samples. (b) Magnified view of the region highlighted by the blue dotted box. (c) Left: refined phase fractions after 5 h current stressing at current densities of 0.5, 0.75, 1.0, 1.5, and 2.0 × 10^3^ A cm^−2^ on η′_6_. Right: refined phase fractions of the time‐resolved η′_6–1e_ to η′_6–5e_ series at fixed 1.5 × 10^3^ A cm^−2^. Phase fractions are normalized to 100 wt.% per sample. Typical refinement statistics are Rwp = 8.6%–9.2%, Rp = 5.5%–8%, and χ^2^ = 3–5.

As shown in Figure 1, when the Cu_6_Sn_5_ samples are subjected to electric currents with a current density of 1.5 × 10^3^ A/cm^2^, the measured average temperature stabilized near 119.76°C ± 0.33°C. To examine whether matched bulk‐temperature exposure alone could reproduce the structural evolution observed under current stressing, we carried out parallel isothermal ageing experiments at 120°C for 1–11 h (η′_1_‐η′_11_). Figure 3 shows that all thermally aged samples retained the reference monoclinic η′ structure within experimental resolution, with no emergence or disappearance of diagnostic features associated with the η phase, confirming that thermal ageing alone does not trigger the η′ → η transformation under these conditions. Thermal ageing primarily influences grain coarsening. As shown in Figure 4a, the average grain size increases from 10.4 ± 0.6 µm (as prepared) to ∼26.2 µm after 4 h (η′_4_), after which it saturates. EBSD IPF maps collected after 6 h (η′_6_) and 11 h (η′_11_) (Figure 4b,c) show no change in grain mapping, indicating that isothermal exposure affects grain size only. For clarity, diagnostic η′ reflections are labelled in Figure 3. These peaks remain unchanged throughout 1–11 h ageing, further confirming structural stability. Although η and η′ share many reflections, η′ exhibits characteristic weak‐multiplet features absent in η; this distinction becomes essential when analyzing current‐stressed samples in Figure 5, where these η′ signatures gradually disappear. As Hough‐based EBSD cannot distinguish η from η′, all EBSD maps are indexed using the η phase library and thus represent η‐phase morphology only. Structural identification (η′ vs. η) is determined exclusively from XRD.

Figure 5 presents the ex situ SR‐XRD patterns of the current‐stressed series at 1.5 × 10^3^ A/cm^2^ for 1–5 h (η′_6–1e_ to η′_6–5e_). Figure 3a provides the laboratory XRD reference (η′_6_) for phase identification, while Figure 5 contains only SR‐XRD patterns; FWHM is therefore evaluated only from the SR‐XRD dataset. The transformation is most apparent in the 31–40° region (Figure 5b). In the early stressed states (η′_6–1e_ and η′_6–2e_), this window shows the characteristic η′ multiplet arising from monoclinic peak splitting. The weak features in this range (including those near ∼33° and ∼38.5°) remain consistent with η′ reflections and are captured by the η′+η GSAS‐II refinements without introducing additional phases (see Figure 5b; Figure S5 for the full‐pattern fit and difference curve). With increasing stress, these multiplet intensities diminish and progressively merge, indicating a rise in symmetry. By 3 h (η′_6–3e_), the multiplet collapses into a single dominant peak at ∼35.05°, corresponding to η(101). Although η′ is still detected quantitatively (Table S2), its distinct signatures in this window are no longer resolvable due to peak overlap and the higher structure‐factor intensity of η(101). This multiplet‐to‐singlet collapse therefore provides a direct crystallographic fingerprint of the η′ → η transformation. The refinements converge with Rwp = 8.6‐9.2%, Rp = 5.5%–8%, and χ^2^ = 3–5, which are typical for Cu_6_Sn_5_ refinements over the restricted 20–55° range. No β‐Sn peak is observed at 2θ ≈ 51.5° [17], and secondary phases are not required in the refinements, placing an upper bound of ∼1 wt.% on any undetected constituent. The small, systematic shift of η(002) within the SR‐XRD series corresponds to a minor d‐spacing change consistent with an evolving strain state during stressing, rather than a new phase Figure 5.

**FIGURE 6:**
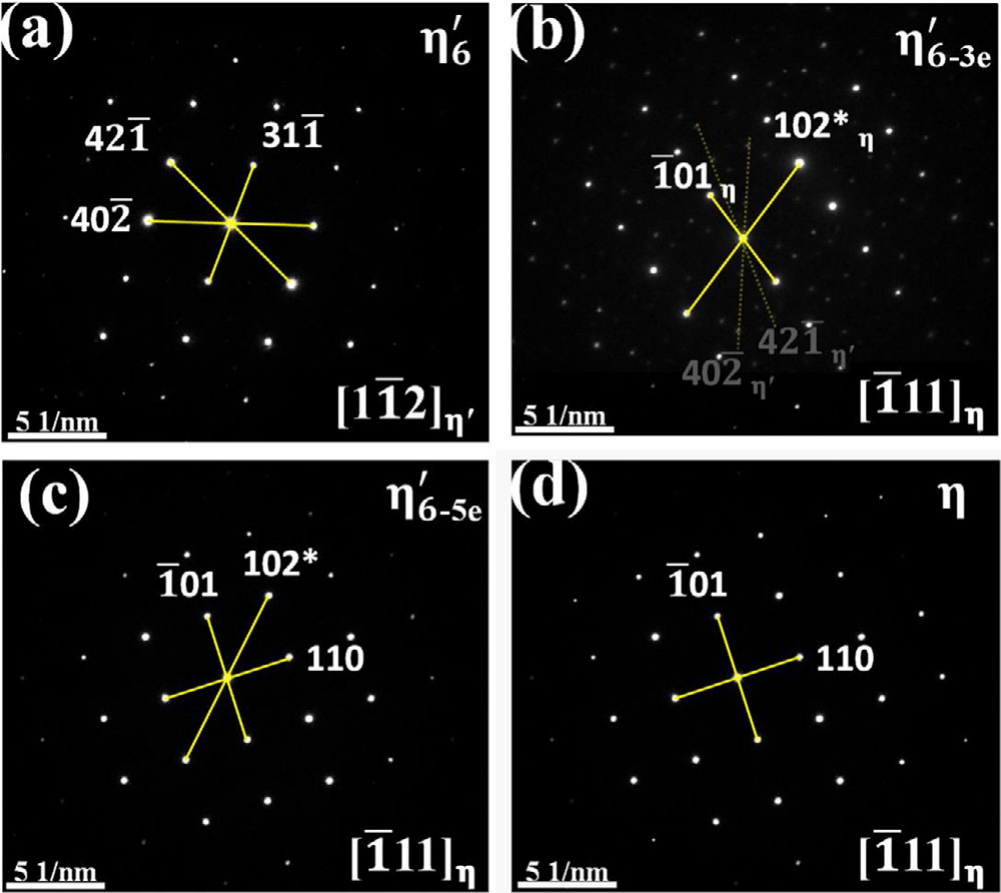
The SAED patterns of (a) η′_6_ (indexed along [$1\bar{1}2$]), (b) η′_6–3e_ (after 3 h current stressing; [$\bar{1}11$]), (c) η′_6–5e_ (after 5 h current stressing; [$\bar{1}11$]), and (d) as‐prepared η‐Cu_6_Sn_5_ (indexed along [$\bar{1}11$]). The asterisk (^*^) marks a weak off‐zone reflection (102)_η_, arising from slight specimen tilt/excitation; it does not affect the indexing or phase assignment.

We conducted TEM analysis to further confirm the monoclinic‐to‐hexagonal phase transition of Cu_6_Sn_5_. Figure 6 shows SAED patterns for η′‐Cu_6_Sn_5_ before current stressing (η′_6_), after 3 h and 5 h at J = 1.5 × 10^3^ A/cm^2^ (η′_6–3e_ and η′_6–5e_), and for the as‐prepared η reference. The SAED pattern in Figure 6a exhibits a diffraction spot indexed to the [$1\bar{1}2$] zone axis of $\eta^{\prime }$. With a 3‐h electric current stressing, a mixed SAED pattern of both monoclinic and hexagonal structure is observed as shown in Figure 6b, which suggests the progress of phase transition. After 5 h of electric current stressing, the diffraction pattern of η′_6–5e_ shows a transformed spot corresponding to [$\bar{1}11$] zone axis of η, as shown in Figure 6c, which is symmetrically similar to the as‐prepared η‐Cu_6_Sn_5_ shown in Figure 6d. The SR‐XRD and TEM/SAED observations in Figures 5 and 6 are mutually consistent and support progressive transformation from the monoclinic η′ structure to the hexagonal η structure under current stressing at a measured bulk temperature of ∼120 °C. At the intermediate 3 h condition, both commensurate η′ superlattice reflections and emerging η reflections are observed, consistent with a partially transformed state. No incommensurate satellite reflections characteristic of η′′ [19] were detected within the angular or reciprocal‐space resolution of the present XRD and SAED measurements.

**FIGURE 7:**
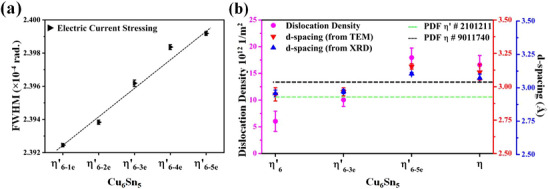
(a) Evolution of the FWHM of the dominant reflection near 2θ ≈ 30° extracted from SR‐XRD profile (Figure 5) fits during electric‐current stressing. In monoclinic states (η′_6–1e_ and η′_6–2e_), the peak is indexed as η′ (11$\bar{3}$), whereas after transformation (η′_6–3e_ to η′_6–5e_) the same 2θ window corresponds to η (101). The dashed line is a guide to the eye. (b) Diagnostic d‐spacing of this reflection obtained from XRD peak positions and TEM/SAED, together with semi‐quantitative projected dislocation areal density estimated from g‐filtered IFFT images. Horizontal dashed lines indicate reference d‐spacings for η′ and η.

Besides the phase transition, we observe systematic changes in peak position (Figure 5a) and refined peak breadth (FWHM; Figure 7a) within the SR‐XRD dataset. Figure 7a tracks the FWHM extracted from SR‐XRD refinements for a diagnostic reflection in the vicinity of 2θ ≈ 30° during current stressing (η′_6‐1e_ to η′_6‐5e_). In the monoclinic η′‐dominated states (η′_6‐1e_ and η′_6‐2e_), this feature is indexed primarily as the (11$\bar{3}$) η′ reflection. After the transformation (η′_6‐3e_ to η′_6‐5e_), the dominant contribution in the same 2θ window is best described by the hexagonal η phase (indexed as (101) η reflection). Because peak widths depend on the instrumental resolution function, FWHM values are compared only within the SR‐XRD series, and the trend is interpreted as the evolution of peak breadth of this diagnostic feature rather than a single‐phase single‐(hkl) quantity. The refined peak breadth increases with stressing time, consistent with progressively increased defect/strain content accompanying the η′ → η transformation.

To track the current‐induced η′ → η transition, we use the dominant reflection near 2θ ≈ 30° as a diagnostic peak: it corresponds to (11$\bar{3}$) in monoclinic η′ (η′_6–1e_ and η′_6–2e_) and to (101) in hexagonal η (η′_6–4e_ to η′_6–5e_). The corresponding d‐spacings extracted from XRD peak positions and TEM‐SAED agree closely (Figure 7b), confirming the phase assignment across techniques. As summarized in Figure 7b and Table S3, the diagnostic spacing lies in the η′ range (∼2.94–2.98 Å) for η′6 and η′6–3e, and shifts to the η range after transformation (∼ 3.10 Å from SR‐XRD and ∼ 3.15 Å from SAED), consistent with reported values for η and η′ [8, 10, 25, 54]. In parallel, semi‐quantitative projected dislocation areal densities estimated from g‐filtered IFFT images increase monotonically (from 6.0 ± 1.9 × 10^12^ m^−2^ (η'_6_) → 10.1 ± 1.3 × 10^12^ m^−2^ (η'_6–3e_) → 18.0 ± 1.8 × 10^12^ m^−2^ (η'_6–5e_), approaching the η benchmark (16.6 ± 1.75 × 10^12^ m^−2^)) with stressing time (Figure 7b; Figure S6). These values are used here only as a comparative indicator consistent with increasing defect accumulation during current stressing, rather than as a primary basis for mechanistic interpretation. Notably, despite the phase transition, EBSD indicates no significant grain growth: the average grain sizes before (η′_6_) and after stressing (η′_6–5e_) are 26 ± 0.6 and 27.2 ± 0.5 µm, respectively (Figure 4b,d).

**FIGURE 8:**
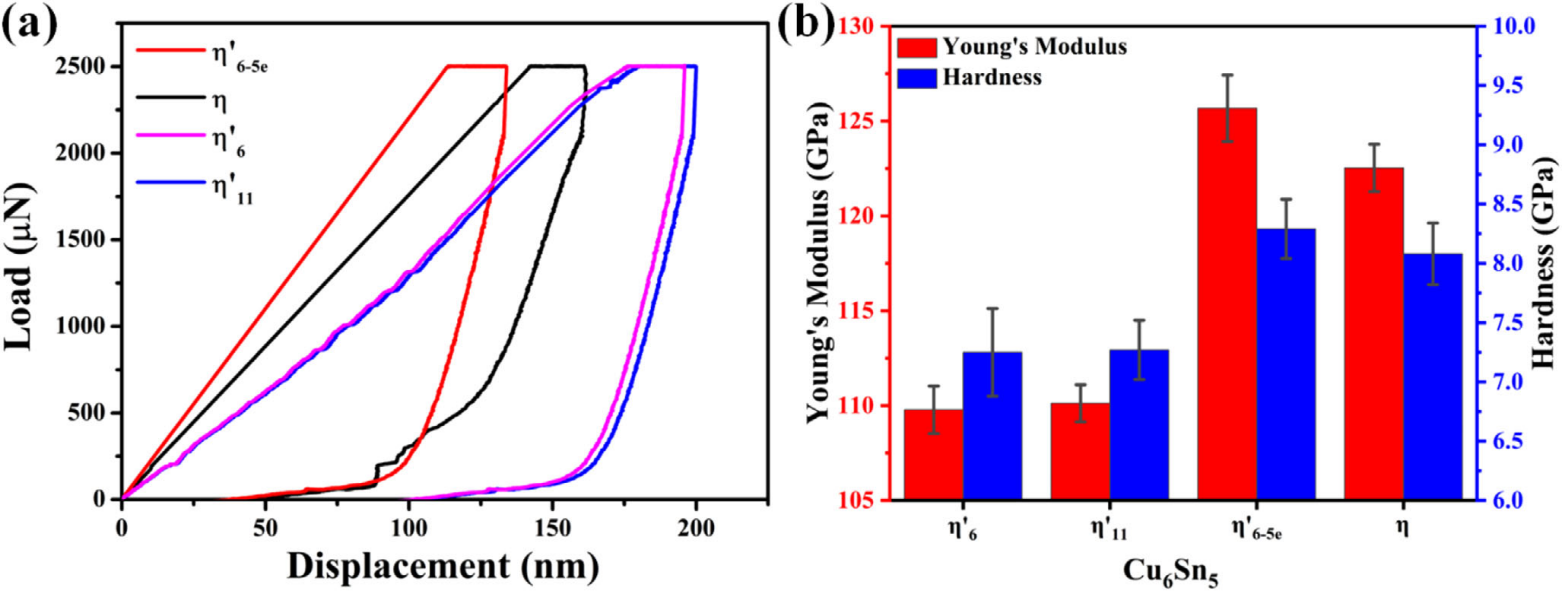
(a) The load–displacement curves and (b) the elastic modulus and hardness obtained from nanoindentation tests for as‐prepared η‐Cu_6_Sn_5_, η′_6_, η′_11,_ and η′_6‐5e_ samples.

Figure 8a shows the load‐displacement curves of $\eta_{6}^{\prime }$, $\eta_{11}^{\prime }$, $\eta_{6-5e}^{\prime }$, and as‐prepared η‐Cu_6_Sn_5_ samples obtained via nanoindentation. The nanoindentation was performed on grains oriented along the [0001] direction with an angular deviation of ±10°. As shown in Figure 8a, the shape of the curves for $\eta_{6}^{\prime }$ and $\eta_{11}^{\prime }$ are very similar, indicating the additional thermal ageing at 120 °C does not significantly change the mechanical properties of the monoclinic Cu_6_Sn_5_. It agrees well with the microstructure observations of similar grain sizes and random orientations as shown in Figure 4. The derived modulus values of the $\eta_{6}^{\prime }$ and $\eta_{11}^{\prime }$ samples are 109.78 ± 1.25 and 110.12 ± 0.98 GPa, respectively, with corresponding hardness values of 7.25 ± 0.87 GPa and 7.18 ± 0.76 GPa, respectively, as shown in Figure 8b. On the other hand, the hexagonal η‐Cu_6_Sn_5_ sample possesses better mechanical properties, with a modulus of 122.53 ± 1.25 GPa and a hardness of 8.08 ± 1.37 GPa, as shown in Figure 8b). A more pronounced change is observed in the η′_6–5e_ sample, where the load–displacement response differs from those of η′_6_ and η′_11_. Under the present test conditions, the η′_6–5e_ sample exhibits a measured indentation modulus of 125.67 ± 1.75 GPa and hardness of 8.29 ± 0.25 GPa. This relative increase is consistent with the current‐induced structural evolution, although the present data do not allow the contributions of phase constitution, defect structure, strain hardening, and possible residual stress to be fully separated.

The mechanical properties of the Cu_6_Sn_5_ IMC with various phase constituents based on different testing methods has been reported in the literature. Emadi et al., reported that the modulus value of Cu_6_Sn_5_ as an IMC (37% hexagonal and 63% monoclinic) was 103.3 ± 0.9 GPa [55], while Deng et al., performed nanoindentation and reported that the modulus value of hexagonal η‐Cu_6_Sn_5_ after ageing at 240 °C was 119 GPa [56]. These values are often referred to as benchmarks for phase‐specific mechanical properties. Previous studies have reported that the modulus of Cu_6_Sn_5_ varies broadly from approximately 84 GPa to 127 GPa, depending on phase composition, crystallographic orientation, and measurement technique [57, 58]. A recent micropillar compression study by Yu et al., on η‐Cu_6_Sn_5_ oriented along the [0001] direction (with a 10° deviation) reported a modulus of 126 ± 2.3 GPa [59]. The elastic modulus and hardness values of Cu_6_Sn_5_ with different phase compositions are summarized in Table S4 of the ESI, which closely match our measured values. This agreement between independent methods underscores the reliability of our measurements and highlights the strong orientation dependence of mechanical response in Cu_6_Sn_5_. While the increase in measured modulus and hardness from η′_6_ to η′_6–5e_ is consistent with the observed current‐induced structural evolution, we do not interpret the slightly higher modulus of η′_6–5e_ as unambiguous evidence of a higher intrinsic elastic stiffness than equilibrium η‐Cu_6_Sn_5_. Rather, η′_6–5e_ is described here as exhibiting a higher measured indentation modulus and hardness under the present test conditions.

## 3 Discussion

Thermal ageing at 120°C for 1–11 h retained the commensurate monoclinic η′ structure and primarily produced grain growth, whereas current stressing under a measured bulk temperature of ∼120°C produced progressive monoclinic‐to‐hexagonal transformation without comparable grain coarsening. Within the present experimental window, matched bulk‐temperature exposure alone did not reproduce the transformation observed under current stressing. This contrast indicates that electric current plays an important role in promoting the observed structural evolution below the equilibrium η′ ↔ η transition temperature. At the same time, the present data do not allow thermal and current‐driven contributions to be fully separated, nor do they exclude possible synergistic interactions among Joule heating, localized defect‐site effects, and current‐induced nonequilibrium processes. We therefore describe the present behavior conservatively as an electric‐current‐induced or current‐assisted monoclinic‐to‐hexagonal transformation occurring under sub‐equilibrium bulk‐temperature conditions. Additional grain‐resolved electrothermal sensitivity analyses are provided in Figure S2; within this continuum grain/grain‐boundary framework, reduced grain‐boundary transport did not generate sustained mesoscale hotspots approaching the equilibrium transition temperature, although atomic‐scale transient heating cannot be excluded by continuum modeling.

The structural observations remain internally consistent. The progressive disappearance of η′ superlattice reflections, the collapse of the monoclinic multiplet into the dominant η(101) feature, the TEM/SAED confirmation of mixed and transformed states, and the increase in refined peak breadth together support the formation of a metastable η‐rich state during current stressing. No evidence of an intermediate incommensurate η′′ state was detected within the angular or reciprocal‐space resolution of the present measurements. The sequential loss of η′ superlattice reflections is also consistent with progressive Cu‐sublattice disordering during the η′ → η order–disorder transition [12, 24], although the present data do not allow that mechanism to be established uniquely. The semi‐quantitative dislocation‐density analysis is likewise consistent with increasing defect accumulation during stressing, but is treated here only as supporting evidence rather than as a primary mechanistic basis.

The current‐transformed η′_6–5e_ state also exhibits higher measured indentation modulus and hardness than the monoclinic reference under the present test conditions. However, we avoid attributing this difference solely to intrinsic lattice stiffening because residual stress, phase constitution, defect structure, and strain hardening were not independently separated. Accordingly, η′_6–5e_ is described here as a retained current‐transformed η‐rich post‐stress state with enhanced measured mechanical response under the present testing conditions. The present work focuses on establishing that electric current can promote and retain unusual phase evolution in Cu_6_Sn_5_ below the equilibrium transition temperature, thereby providing an experimental basis for understanding current‐affected phase stability in conductive intermetallics.

## 4 Conclusion

In summary, Cu_6_Sn_5_ exhibits an electric‐current‐induced monoclinic‐to‐hexagonal transformation under a measured bulk temperature of ∼120 °C, below the equilibrium η′ ↔ η transition temperature. Ex situ synchrotron XRD of separately current‐stressed samples together with TEM/SAED shows progressive conversion from η′ to η during current stressing, whereas matched bulk‐temperature thermal ageing alone did not reproduce the same transformation within the examined time window. The ex situ diffraction results therefore indicate retention of an η‐rich post‐stress state formed under sub‐equilibrium bulk‐temperature conditions. Under the present test conditions, this state also exhibits a higher measured indentation modulus and hardness than the monoclinic reference. These findings show that electric current can promote unconventional phase evolution in Cu_6_Sn_5_ and provide a useful experimental basis for future studies of current‐assisted phase stability in conductive intermetallic compounds.

## 5 Experimental Details

### 5.1 Sample Preparations

Cu_6_Sn_5_ (Sn = 60.9 wt.%) samples were synthesized using an arc melting furnace (MAM1 Arc Melter, Edmund Buhler, Germany) under an Ar atmosphere. Each ingot was prepared from ∼1.5–2 g of high‐purity Cu (99.9%, Alfa Aesar, USA) and Sn (99.8%, Alfa Aesar, USA) pellets in the stoichiometric ratio. This sample size was selected to ensure homogeneous melting and rapid quenching while providing sufficient material for subsequent annealing and following characterizations. The obtained alloy was transferred into a vacuum quartz tube (∼10^−5^ Pa) for subsequent heat treatments. One batch of as‐arc samples was annealed at 150°C for 24 h in a vertical furnace, while the other batch underwent the same process at 300°C for 24 h to fabricate low‐temperature monoclinic η′‐Cu_6_Sn_5_ and high‐temperature hexagonal η‐Cu_6_Sn_5_, respectively. The as‐prepared monoclinic and hexagonal reference samples are denoted as η′ and η, respectively. Following annealing, the alloys were rapidly quenched in water at room temperature (RT). The as‐prepared η′‐Cu_6_Sn_5_ samples were then further annealed at 120°C under vacuum for 1–6 h, denoted η′_1_ to η′_6_, in order to homogenize the grain size prior to the comparison experiments. The ageing temperature of 120°C was selected based on the measured average sample temperature reached during electric‐current stressing, as described below.

The setup shown in Figure 1b was used to apply electric current to the 6 h pre‐annealed η′‐Cu_6_Sn_5_ samples (η′_6_) using a power supply (Keithley 3500, Keithley, USA). The specimen geometry and current direction are shown in Figure 1c (3.5 mm × 3.5 mm × 60 µm). A current density of 1.5 × 10^3^ A cm^−2^ was selected as the principal condition for the time‐resolved study. This value lies at the lower end of the 10^3^–10^4^ A cm^−2^ range commonly used in Cu–Sn current‐stressing studies [60, 61], and preliminary current‐density mapping at J = 0.5, 0.75, 1.0, 1.5, and 2.0 × 10^3^ A cm^−2^ (5 h each; Figure S1) showed that lower current densities (≤ 1 × 10^3^ A cm^−2^) did not produce near‐complete transformation within the 5 h experimental window [62, 63].

The temperature profile during current stressing was monitored using a K‐type thermocouple attached to the sample surface with non‐conductive glue, as shown in Figure 1b. The recorded temperature profiles are shown in Figure 1d. For the time‐resolved series, five separate η′_6_ samples were each subjected to continuous direct current (DC) stressing for 1, 2, 3, 4, and 5 h, respectively, denoted η′_6–1e_, η′_6–2e_, η′_6–3e_, η′_6–4e_, and η′_6–5e_. The resulting stressed samples were then characterized ex situ by SR‐XRD after current removal. Electric‐current stressing was applied at the synchrotron beamline. After stressing, the current was switched off, and each sample was allowed to cool to equilibrium room temperature before SR‐XRD acquisition, so that all diffraction scans were collected under the same post‐stress scanning conditions. Accordingly, the SR‐XRD patterns reported here represent retained post‐stress states after current removal. During the current application, the measured equilibrated sample temperatures ranged from 119.50°C to 120.15°C, with an average of 119.76°C ± 0.33°C.

After establishing η′_6_ as the common starting state, two parallel post‐treatment branches were used for comparison. In one branch, separate η′_6_ samples were subjected to continuous DC stressing in ambient atmosphere for 1–5 h, as described above. In the other branch, separate η′_6_ samples were subjected to isothermal exposure in ambient atmosphere at 120°C for the corresponding durations without current, denoted η′_7_ to η′_11_. This post‐η′_6_ design enabled direct comparison between ambient current stressing and ambient matched bulk‐temperature thermal exposure, with the presence or absence of electric current as the primary controlled variable. The detailed nomenclature is listed in Table 1, and the experimental branching sequence is summarized schematically in Figure S3.

**TABLE 1:** Nomenclature of the Cu_6_Sn_5_ at its different conditions with thermal annealing and electric current stress.

Condition	Sample ID
Monoclinic Cu_6_Sn_5_ prepared by annealing as‐cast sample at 150°C for 24 h	η′
Hexagonal Cu_6_Sn_5_ prepared by annealing as‐cast sample at 300°C for 24 h	η
120°C thermal annealing upon as‐prepared monoclinic Cu_6_Sn_5_ (η′) samples for 1, 2, 3, 4, 5, 6, 7, 8, 9, 10, and 11 h	$\eta_{1}^{\prime }$, $\eta_{2}^{\prime }$, $\eta_{3}^{\prime }$, $\eta_{4}^{\prime }$, $\eta_{5}^{\prime }$, $\eta_{6}^{\prime }$, $\eta_{7}^{\prime }$, $\eta_{8}^{\prime }$, $\eta_{9}^{\prime }$, $\eta_{10}^{\prime }$, $\eta_{11}^{\prime }$
1.5 × 10^3^ A/cm^2^ electric current stressing upon 120°C and 6‐h pre‐annealed monoclinic Cu_6_Sn_5_ ($\eta_{6}^{\prime }$) for 1, 2, 3, 4, and 5 h.	$\eta_{6-1e}^{\prime }$, $\eta_{6-2e}^{\prime }$ $\eta_{6-3e}^{\prime }$, $\eta_{6-4e}^{\prime }$, $\eta_{6-5e}^{\prime }$

### 5.2 Sample Characterizations

Powder X‐ray diffraction (PXRD) was first used to identify the as‐prepared η′‐Cu_6_Sn_5_ and η‐Cu_6_Sn_5_ phases. For these phase‐identification scans, the samples were ground into powders with an average particle size <10 µm and measured on a Bruker D8 Advance diffractometer using Cu Kα radiation (λ = 1.5406 Å) in Bragg–Brentano geometry. Data were collected over 20–55° (2θ) with a step size of 0.013° and a scan rate of 4°/min. All XRD measurements up to the isothermal‐annealing series were performed using the laboratory diffractometer. In contrast, only the current‐stressed bulk samples (η′_6–1e_, η′_6–2e_, η′_6–3e_, η′_6–4e_, and η′_6–5e_) were characterized by synchrotron X‐ray diffraction (SR‐XRD) to resolve weak diagnostic reflections of η′‐Cu_6_Sn_5_ and enable quantitative profile analysis. These synchrotron measurements were performed ex situ on separately stressed samples after the current had been switched off. SR‐XRD was conducted at NSRRC (TLS 17A1, 8 keV; λ = 1.55 Å) with a 0.002° step size. Peak‐breadth (FWHM) values reported in this work were extracted from SR‐XRD refinements and are compared only within the SR‐XRD dataset. Synchrotron patterns were converted to Cu Kα‐equivalent 2θ for comparison with COD reference cards (PDF #2101211 for η′ and #9011740 for η). Rietveld analysis was performed using GSAS‐II (v5.6.1). The refined parameters included scale factors, lattice parameters (a, c for η; a, b, c, β for η′), background coefficients (Chebyshev‐1, 6 terms), zero‐shift, and instrumental peak‐broadening parameters (U, V, W). Atomic coordinates and microstructural terms (preferred orientation, crystallite size, microstrain) were kept fixed to avoid over‐parameterization of the heavily overlapping η/η′ reflections. The refinements converged with Rwp = 8.6–9.2%, Rp = 5.5%–8%, and χ^2^ = 3–5, values that are typical for multiphase Cu‐Sn intermetallics refined over a limited 2θ range.

We performed scanning electron microscopy (SEM, SU3500, Hitachi, Japan) equipped with energy dispersive spectroscopy (EDS, EDAX Inc., USA) to observe the sample surface morphology and composition. The measurements were conducted in both secondary‐electron (SE) and backscattered‐electron (BSE) modes under the operating conditions: accelerating voltage = 15 kV (for both imaging and EDS), probe current = 5 nA, and working distance ≈ 10 mm (for EDS acquisition). BSE images provided compositional contrast. Additionally, electron backscattered diffraction (EBSD, Hikari, EDAX, USA) mapping was performed on mechanically polished surfaces finished with colloidal silica. EBSD maps were acquired using conventional Hough‐based indexing, and diffraction patterns were indexed using only the η‐Cu_6_Sn_5_ hexagonal structure (P6_3_/mmc). Because conventional Hough‐based EBSD cannot reliably distinguish η from η′ due to their nearly identical Kikuchi‐band geometry, all successfully indexed points were assigned to η‐Cu_6_Sn_5_. The reported IPF maps are referenced to the sample normal direction (ND), i.e., the surface normal of the mapped plane. To ensure robust orientation statistics, pixels with the confidence index (CI) < 0.10 and/or Fit > 2° were discarded from subsequent analysis and plotted as non‐indexed in the maps. Regions of low image quality (e.g. pores, pull‐outs, or grain/pore boundaries) generated weak or distorted patterns, resulting in low CI values; these appear as dark areas in the IQ maps (Figure 2b,e) and were treated as non‐indexed in all EBSD‐based quantifications. Grain size was extracted directly from EBSD maps, and phase fractions for the current‐stressed series were determined from SR‐XRD Rietveld refinements. Selected‐area electron diffraction (SAED) and high‐resolution TEM (HR‐TEM) were acquired on a JEOL JEM‐2100F (Japan). TEM foils were prepared by FIB (FEI NOVA 200, Thermo Fisher, USA). To quantify dislocations, each HR‐TEM image was Fourier‐transformed (FFT); a single strong reflection of the imaged phase was selected together with its Friedel pair (g) as guided by SAED. A circular mask was applied at g (all other frequencies suppressed), and the inverse Fourier‐transformed (IFFT) yielded a g‐filtered lattice‐fringe image in which dislocation cores appear as fringe terminations/phase discontinuities. For each condition, 8–12 non‐overlapping windows of known area were selected from thin, uniform‐contrast regions, and dislocation cores were manually annotated on the HR‐TEM images. We conducted nanoindentation tests using a Berkovich indenter tip (PI 88, Bruker, USA) in load‐controlled mode to determine the elastic modulus. A maximum load of 2500 µN was applied with a loading/unloading rate of 200 µN/s, and a holding time of 5 s at peak load was maintained to minimize creep effects. The measurements were performed on grains oriented along the [0001] direction, with an angular deviation of ±10°, as confirmed by EBSD prior to indentation.

## Author Contributions

**Shih‐kang Lin**: Conceptualization, Resources, Supervision, Project administration, Funding acquisition, Writing review & editing. Shubhayan Mukherjee: Investigation, Methodology, Formal analysis, Data curation, Writing original draft. **Yu‐chen Liu**: Validation, Writing review & editing. **Jun Mizuno**: Supervision.

## Funding

This research was funded by the National Science and Technology Council (NSTC) in Taiwan (114‐2628‐E‐006‐003, 114‐2923‐E‐006‐004, 114‐2622‐8‐006‐016, and 114‐2923‐E‐006‐001). This work was also partially supported by the Hierarchical Green‐Energy Materials (Hi‐GEM) Research Center, from the Featured Areas Research Center Program within the framework of the Higher Education Sprout Project by the Ministry of Education (MOE) in Taiwan.

## Conflicts of Interest

The authors declare no conflicts of interest.

## Supporting information




**Supporting File**: advs75499‐sup‐0001‐SuppMat.docx.

## Data Availability

All data that supports the conclusions of the paper have been presented in the manuscript and supplementary information.
