# Supplementary material for: Electric‐Current‐Induced Phase Transformation in Cu6Sn5 Below Its Equilibrium Transition Temperature
Source: Adv Sci (Weinh). 2026 May 8;13(41):e75499. doi: 10.1002/advs.75499 (PMC13335739; doi:10.1002/advs.75499)
Supplement: Supplementary file 1 — Supporting File: advs75499‐sup‐0001‐SuppMat.docx. [file ADVS-13-e75499-s001.docx]

**Electric-Current-Induced Phase Transformation in Cu_6_Sn_5_ Below Its Equilibrium Transition Temperature**

Shih-kang Lin^1-4,*^, Shubhayan Mukherjee^1^, Yu-chen Liu^4-5^, and Jun Mizuno^4^

^1^ Department of Materials Science and Engineering, National Cheng Kung University, Tainan 70101, Taiwan;

^2^ Core Facility Center, National Cheng Kung University, Tainan 70101, Taiwan;

^3^ Center for Resilience and Intelligence on Sustainable Energy Research (RiSER), National Cheng Kung University, Tainan 70101, Taiwan;

^4^ Academy of Innovative Semiconductor and Sustainable Manufacturing, National Cheng Kung University, Tainan 70101, Taiwan;

^5^ Department of Mechanical Engineering, National Cheng Kung University, Tainan 70101, Taiwan.

[*linsk@mail.ncku.edu.tw](mailto:*linsk@mail.ncku.edu.tw)

# **Supplementary Data**


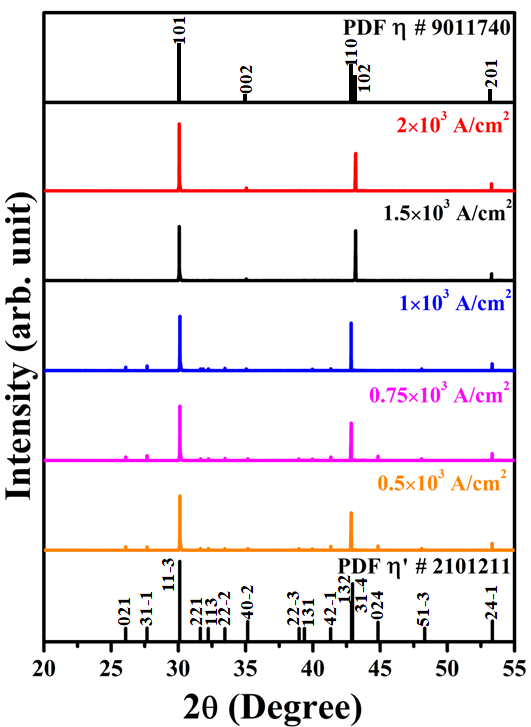


**Fig. S1.** SR-XRD peak profile of the $\eta’$-phase under the current density from 0.5 to 2$\times$10^3^ A/cm^2^ for 5 h [1-2].

The electrothermal field in the η′-Cu_6_Sn_5_ sample (3.5 mm × 3.5 mm × 60 µm) during current stressing was simulated using finite element analysis (FEA) in COMSOL Multiphysics. The baseline continuum model assumed an applied current density of 1.5 × 10^3^ A cm^-2^ for 5 h in air, with natural convection to the surroundings (heat-transfer coefficient $h=20$W m^-2^ K^-1^), an ambient temperature of 32 °C, and surface-to-ambient radiation with an emissivity of 0.9. Bulk thermal and electrical properties of Cu_6_Sn_5_ were used for this homogeneous model, which served to evaluate the sample-scale spatial uniformity of the electrothermal field. To further test whether microstructural heterogeneity could generate sustained mesoscale hotspots, additional grain-resolved sensitivity analyses were performed using explicit grain and grain-boundary domains. In polycrystalline systems, grain-boundary scattering is known to produce substantial differences between grain-boundary and bulk transport properties. The classical Mayadas–Shatzkes model shows that grain-boundary scattering can strongly increase electrical resistivity in polycrystalline films, and Zhang *et al.* reported that both electrical and thermal conductivities in polycrystalline Au nanofilms are strongly reduced by grain-boundary scattering [3-4]. Because corresponding thermal and electrical transport properties for Cu_6_Sn_5_ grain-boundary domains are not available in the literature, the grain-boundary thermal conductivity and electrical conductivity were treated as sensitivity parameters and represented as scaled fractions of the corresponding bulk Cu_6_Sn_5_ values according to $k_{GB}=f_{k}k_{bulk}$ and $\sigma_{GB}=f_{\sigma}\sigma_{bulk}$, where $k_{GB}$ is the grain-boundary thermal conductivity, $f_{k}$ is the dimensionless scaling factor for grain-boundary thermal transport, $k_{bulk}$ is the bulk thermal conductivity of Cu_6_Sn_5_, $\sigma_{GB}$ is the grain-boundary electrical conductivity, $f_{\sigma}$ is the dimensionless scaling factor for grain-boundary electrical transport, and $\sigma_{bulk}$ is the bulk electrical conductivity of Cu_6_Sn_5_. Two bounding sensitivity cases were examined: a bulk-equivalent grain-boundary case with $f_{k}=f_{\sigma}=1$, and a weakened grain-boundary transport case with $f_{k}=f_{\sigma}=0.2$. The latter was adopted as a bounding sensitivity case motivated by the strong grain-boundary transport suppression reported in other polycrystalline systems, although it is not intended as a measured Cu_6_Sn_5_ grain-boundary property. These simulations were used as sensitivity analyses for mesoscale temperature localization rather than as exact quantitative reproductions of the measured thermocouple temperature. In the grain-resolved steady-state cases, the temperature field remained highly uniform, with total spatial variations of only ~0.12–0.14 °C across the sample, and the simulated local maxima remained far below the equilibrium η′ ↔ η transition temperature of 186 °C.


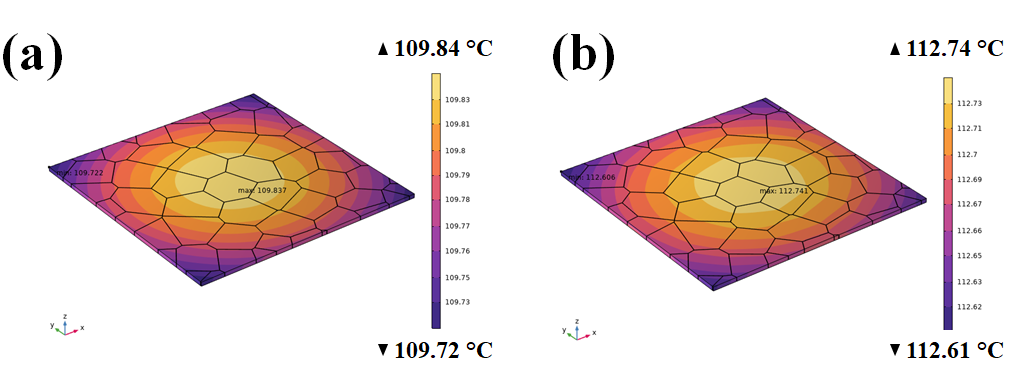


**Fig. S2.** Finite element analysis (FEA) of the electrothermal field in Cu_6_Sn_5_ during current stressing. (a) Grain-resolved steady-state sensitivity analysis with grain-boundary transport represented by $f_{k}=f_{\sigma}=1$. (b) Grain-resolved steady-state sensitivity analysis with weakened grain-boundary transport represented by $f_{k}=f_{\sigma}=0.2$. In both grain-resolved cases, the steady-state temperature field remains highly uniform at the continuum grain scale, and the simulated local temperatures remain far below the equilibrium η′ ↔ η transition temperature.


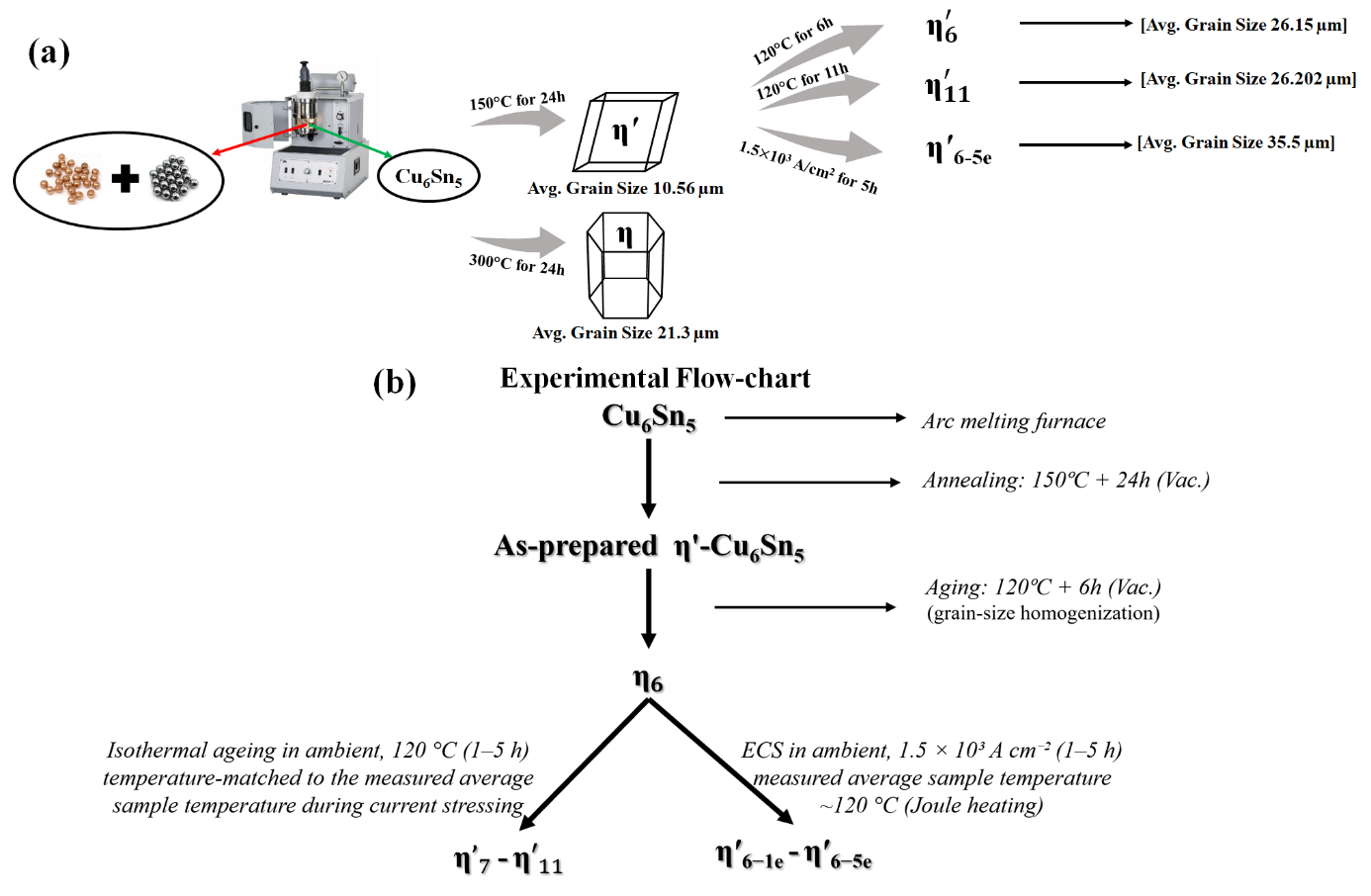


**Fig. S3.** (a) Schematic summary of the preparation route and representative post-treatment states of Cu_6_Sn_5_, including the as-prepared η′ and η phases and selected comparison states together with their average grain sizes. (b) Experimental flowchart illustrating the sample history used in this work. Arc-melted Cu_6_Sn_5_ was first annealed at 150 °C for 24 h to obtain as-prepared η′-Cu_6_Sn_5_. This η′ state was then vacuum-aged at 120 °C to reach the common starting state η′_6_, after which the samples were divided into two parallel branches: ambient isothermal ageing at 120 °C without current (η′_7_ – η′_11_) and ambient electric-current stressing at 1.5 × 10^3^ A cm^−2^ (η′_6−1e_ – η′_6−5e_).


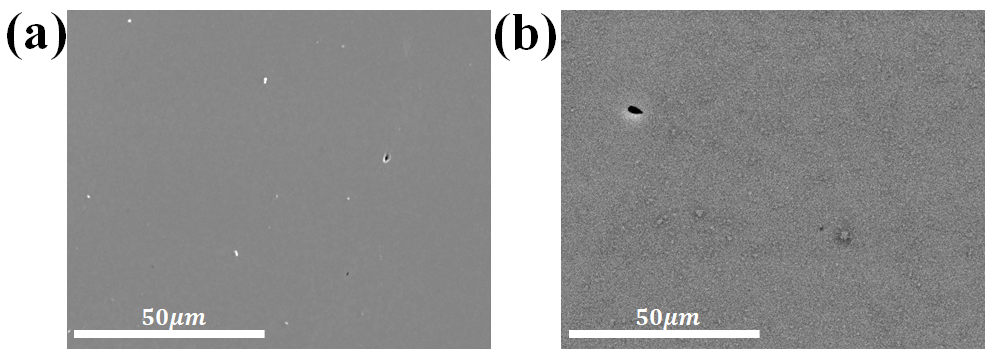


**Fig. S4.** Backscattered-electron images (BEIs) of the as-prepared (a) η′-Cu_6_Sn_5_ and (b) η-Cu_6_Sn_5_ phases, showing dense Cu_6_Sn_5_ microstructures without continuous second-phase regions. Dark contrast features mainly arise from pores/pull-outs and surface topography rather than a distinct phase.


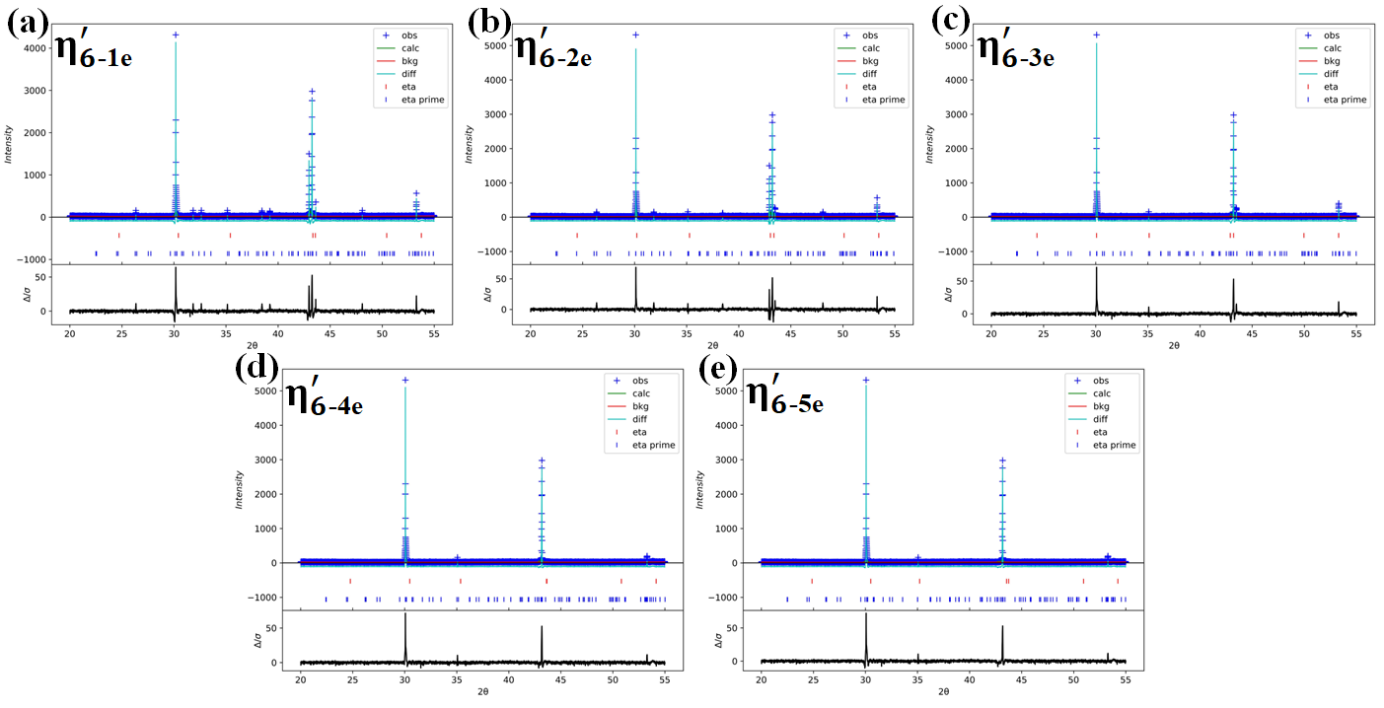


**Fig. S5.** Rietveld refinement of SR-XRD patterns for Cu_6_Sn_5_ samples subjected to electric-current stressing for (a) 1 h, (b) 2 h, (c) 3 h, (d) 4 h, and (e) 5 h. Blue crosses represent the observed intensity, the green line is the calculated profile, the red line is the refined background, and the cyan line (offset) is the difference (I_obs_ − I_calc_). Tick marks indicate allowed Bragg positions for η and η′ phases. The refinements were performed in GSAS-II using a pseudo-Voigt profile function with refinable background, peak-shape, scale factor, and lattice parameters for both η and η′ phases.

Rietveld refinements were carried out in GSAS-II (v5.6). The background was modeled using a 6-term Chebyshev polynomial. Peak shapes were fitted using a pseudo-Voigt profile with refinable U, V, W, and SH/L parameters. The refined parameters included scale factors, zero shift, background coefficients, instrumental peak-shape parameters, and lattice parameters for both η and η′ phases.

For each HR-TEM field, a strong reflection g and its Friedel pair were selected (from SAED/FFT of the same region), circularly white masked, and IFFT-filtered to reveal fringe terminations/phase discontinuities as dislocation cores. Per condition, 8-12 non-overlapping windows from thin, uniform-contrast areas were analyzed. This metric is comparative and intended to track the monotonic trend; absolute values depend on imaging/contrast and are not claimed.


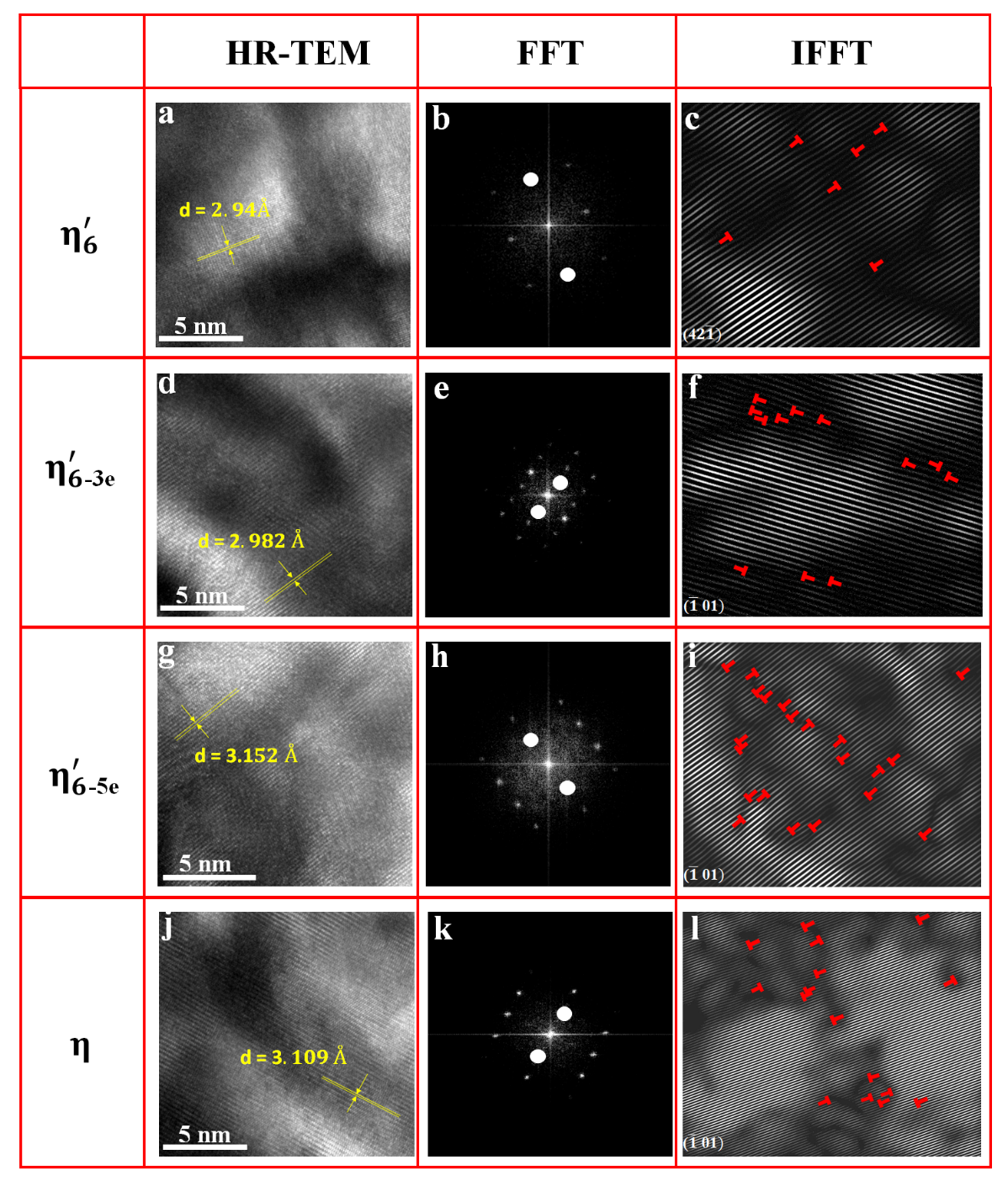


**Fig. S6.** HRTEM–FFT–IFFT triplets from identical fields of Cu₆Sn₅ showing interplanar spacings and dislocation features before and after current stressing. Left column: HRTEM with measured “d" (yellow). Middle: FFTs with white circular masks marking the ±g reflections used for filtering. Right: g-filtered IFFTs from the same regions; red ⊣ markers denote dislocation cores counted for the projected areal density. Rows: (a–c) η′_6_, (d–f) η′_6–3e_, (g–i) η′_6–5e_, and (j–l) as-prepared η-Cu_6_Sn_5_. The g-vector used for each IFFT is indicated on the panel. (Off-zone weak spots, when present, were not used for counting and do not affect phase assignment.)

**Table S1:** Sample compositions of the as-prepared Cu_6_Sn_5_ from EDS analysis.

| η-Cu_6_Sn_5_ | wt% | at% | η′-Cu_6_Sn_5_ | wt% | at% |
| --- | --- | --- | --- | --- | --- |
| Cu | 39.08 | 54.51 | Cu | 39.1 | 54.52 |
| Sn | 60.92 | 45.49 | Sn | 60.90 | 45.47 |

**Table S2:** Phase fractions (wt.%) of η and η′ in Cu_6_Sn_5_ during electric-current stressing (η′_6–1e_ → η′_6–5e_), obtained from GSAS-II Rietveld refinements of the SR-XRD data (reported as 2θ shown as Cu Kα-equivalent for comparison). All phase fractions are normalized to 100 wt.% per sample. Based on the refinement statistics (Rwp = 8.6-9.2 %, Rp = 5.5-8 %, χ^2^ = 3-5), the uncertainty in the extracted phase fractions is estimated to be within ±0.5-1 wt.%. Full refinement profiles are provided in Fig. S5.

| Condition of Cu_6_Sn_5_ | Phase Fraction (wt.%) | |
| --- | --- | --- |
|  | η-phase | η′-phase |
| $\eta_{6-1e}^{'}$ | 0.68 | 99.32 |
| $\eta_{6-2e}^{'}$ | 7.90 | 92.10 |
| $\eta_{6-3e}^{'}$ | 52.31 | 47.69 |
| $\eta_{6-4e}^{'}$ | 95.35 | 4.65 |
| $\eta_{6-5e}^{'}$ | 99.22 | 0.78 |

**Table S3:** Diagnostic d-spacings of the η'(11$\bar{3}$) and η(101) reflection and projected dislocation densities of Cu_6_Sn_5_. d-spacings are given from XRD and TEM/SAED for the same reflection plane used in Fig. 7b. Dislocation densities are projected areal values from g-filtered IFFT images (semi-quantitative).

| Condition of Cu_6_Sn_5_ | Reflection (hkl) | d-spacing (Å)  (TEM/SAED) | d-spacing (Å)  (XRD) | Dislocation Density ($\times$10^12^) (1/m^2^) | Ref. |
| --- | --- | --- | --- | --- | --- |
| η (ref.)  η' (ref.)  $\eta_{6}^{'}$  $\eta_{6-3e}^{'}$  $\eta_{6-5e}^{'}$  η | (101)_Hex._  (11$\bar{3}$)_Mono._  (11$\bar{3}$)_Mono._  (11$\bar{3}$)/(101)^*^  (101)_Hex._  (101)_Hex._ | -  -  2.943 ± 0.05  2.982 ± 0.03  3.152 ± 0.02  3.109 ± 0.06 | 3.10-3.11  ~2.98  2.958 ± 0.025  2.972 ± 0.015  3.105 ± 0.01  3.073 ± 0.03 | -  -  6.035 ± 1.9  10.053 ± 1.26  17.95 ± 1.8  16.598 ± 1.75 | Furtauer et al. [5]  Yu et al. [6]  This work  This work  This work  This work |

** Diagnostic peak.*

**Table S4.** Elastic modulus and hardness values of Cu_6_Sn_5_ with different phase compositions.

| Structure of Cu_6_Sn_5_ | Young’s Modulus (GPa) | Hardness (GPa) | Ref. |
| --- | --- | --- | --- |
| Cu_6_Sn_5_ in Cu/Sn/Cu SLID  (37% of Hexagonal and 63% of Monoclinic) | 113.6 ± 1.1 | 6.7 ± 0.5 | Emadi *et al.* [7] |
| Thermally aged Cu_6_Sn_5_ as IMC in Sn–3.5Ag/Cu (Hexagonal)  Micropillar compression of  η-Cu_6_Sn_5_ ([8] at $\varphi=21^{\circ}$) | 112.3 ± 1.1  126 ± 2.3 | 6.38± 0.21  - | Deng *et al.* [9]  Yu *et al.* [10] |
| $\eta_{6}^{'}$  $\eta_{11}^{'}$ | 109.78 ± 1.25  110.12 ± 0.98 | 7.25 ± 0.87  7.27 ± 0.28 | This work  This work |
| $\eta_{6-5e}^{'}$ | 125.67 ± 1.75 | 8.29 ± 0.25 | This work |
| η-Cu_6_Sn_5_ | 122.53 ± 1.25 | 8.08 ± 1.37 | This work |

References

[1] S. Mukherjee, S.-k. Lin, Impact of Electrical Current Stressing on η'-Cu 6 Sn 5 in Electronics Packaging, in *2024 19th International Microsystems, Packaging, Assembly and Circuits Technology Conference (IMPACT)* IEEE, **2024**, 197-200.

[2] S. Mukherjee, Y.-C. Liu, S.-K. Lin, Phase Transitions of the CU 6 Sn 5 Phase Under Electric Currents, in *2025 International Conference on Electronics Packaging and iMAPS All Asia Conference (ICEP-IAAC)* IEEE, **2025**, 95-96.

[3] A. Mayadas, M. Shatzkes, Electrical-resistivity model for polycrystalline films: the case of arbitrary reflection at external surfaces *Physical review B* **1970**, *1* (4), 1382.

[4] Q. Zhang, B. Cao, X. Zhang, M. Fujii, K. Takahashi, Influence of grain boundary scattering on the electrical and thermal conductivities of polycrystalline gold nanofilms *Physical Review B—Condensed Matter and Materials Physics* **2006**, *74* (13), 134109.

[5] S. Fürtauer, D. Li, D. Cupid, H. Flandorfer, The Cu–Sn phase diagram, Part I: new experimental results *Intermetallics* **2013**, *34*, 142.

[6] C. Yu, J. Liu, H. Lu, P. Li, J. Chen, First-principles investigation of the structural and electronic properties of Cu6− xNixSn5 (x= 0, 1, 2) intermetallic compounds *Intermetallics* **2007**, *15* (11), 1471.

[7] F. Emadi, V. Vuorinen, G. Ross, M. Paulasto-Kröckel, Co, In, and Co–In alloyed Cu6Sn5 interconnects: Microstructural and mechanical characteristics *Materials Science and Engineering: A* **2023**, 145398.

[8] I. H. Bell, U. K. Deiters, On the construction of binary mixture p‐x and T‐x diagrams from isochoric thermodynamics *AIChE Journal* **2018**, *64* (7), 2745.

[9] X. Deng, N. Chawla, K. Chawla, M. Koopman, Deformation behavior of (Cu, Ag)–Sn intermetallics by nanoindentation *Acta materialia* **2004**, *52* (14), 4291.

[10] J. Yu, J. Wu, L. Yu, H. Yang, C. Kao, Micromechanical behavior of single-crystalline Cu 6 Sn 5 by picoindentation *Journal of Materials Science* **2017**, *52*, 7166.
